# OLDER ADULTS WITH MULTIPLE CHRONIC CONDITIONS FROM VULNERABLE POPULATIONS: THE SUCCESS OF PATIENT PRIORITIES CARE

**DOI:** 10.1093/geroni/igad104.1418

**Published:** 2023-12-21

**Authors:** Rafael Samper-Ternent, Lee Jennings

## Abstract

As older adults in the USA grow more diverse, equitable translation of knowledge and best practices to vulnerable groups is essential. Older adults with multiple chronic conditions (MCC) benefit from care focused on the health priorities of individuals. Health priorities include: 1) core values that provide meaning to life; 2) specific, actionable, and realistic health goals that reflect these values; and 3) care preferences that include what individuals are able and willing to do (or not) to achieve their goals. For vulnerable older adults, adverse life experiences provide context to their values. A diverse panel will review the Patient Priorities Care (PPC) approach and describe how PPC has been successfully adapted at different institutions. The first presentation will describe PPC and the importance of identifying values, goals, and health preferences for older adults with MCC. The presenter will show that PPC improves outcomes among older adults, their families, and healthcare providers. The second presentation will describe the implementation of PPC in the VA system. The presenter will describe the impact of PPC and efforts to disseminate it. The third and fourth presentations will describe the cultural adaptation of PPC for older African Americans and older Hispanics respectively. The presenters will describe the adaptation process and discuss the barriers they faced, and the lessons learned from each population group. The panel will conclude by providing suggestions on how to disseminate and increase the adoption of PPC to identify what matters most for diverse older adults.

